# Peptidomics screening for the discovery of uterotonic plant peptides

**DOI:** 10.1186/2050-6511-13-S1-A89

**Published:** 2012-09-17

**Authors:** Johannes Koehbach, Alfred F Attah, Margaret O’Brien, Michael Freissmuth, Christian W Gruber

**Affiliations:** 1 Center for Physiology and Pharmacology, Medical University of Vienna, 1090 Vienna, Austria; 2 Department of Pharmacognosy, Faculty of Pharmacy, University of Ibadan, Ibadan, Oyo State, Nigeria; 3 National University of Ireland, National Center for Biomedical and Engineering Science, Galway, Ireland

## Background

Drug discovery from natural products is still one of the biggest sources of novel lead compounds. In particular, plant cyclotides, disulfide-rich peptides comprising three conserved disulfide bonds in a knotted arrangement, known as cyclic cystine knot motif, and a head-to-tail cyclization, have been extensively investigated over the last four decades for their use as scaffolds in drug development. However, their distribution among flowering plants still remains limited to few species of the families of *Rubiaceae* (coffee), *Violaceae* (violet), *Cucurbitaceae* (cucurbit), *Fabaceae* (bean) and recently *Solanaceae* (potato family), but it is very likely that cyclotides are more widely distributed since their predicted number in *Rubiaceae* alone is ~50.000. Additionally, the pharmacological validation of plants used in traditional medicines may trigger the discovery of novel uterotonic compounds [1].

## Methods and results

Based on the use of plants in traditional Nigerian medicine during pregnancy and childbirth, we analyzed several plants from different families to evaluate their uterotonic properties at cellular level and to identify cyclotides as active molecules. Using a MALDI-TOF/TOF-based screening protocol we were able to identify many novel cyclotide-containing species which was confirmed by manual *de novo* sequencing and automated database identification. The aqueous extracts and semipure peptide fractions have been tested further in a collagen gel contractility assay model and showed varying ability to induce contractions in human myometrial smooth muscle cells.

## Conclusions

In conclusion, our results underpin earlier suggestions that cyclotides are one of the largest peptide classes within plants, covering a large chemical space based on their high sequence diversity. The evaluation of contractile properties of plants used in traditional medicines offers new starting points for the discovery and development of peptide-based uterotonic drug leads.
